# Remote Ischemic Postconditioning vs. Physical Exercise After Stroke: an Alternative Rehabilitation Strategy?

**DOI:** 10.1007/s12035-021-02329-6

**Published:** 2021-02-24

**Authors:** Xiaokun Geng, Qingzhu Wang, Hangil Lee, Christian Huber, Melissa Wills, Kenneth Elkin, Fengwu Li, Xunming Ji, Yuchuan Ding

**Affiliations:** 1 Department of Neurology and China-America Institute of Neuroscience, Xuanwu Hospital, Capital Medical University, Beijing, 101149 China; 2 China-America Institute of Neuroscience, Luhe Hospital, Capital Medical University, Beijing, China; 3 Department of Neurology, Beijing Luhe Hospital, Capital Medical University, Beijing, China; 4 Department of Neurosurgery, Wayne State University School of Medicine, Detroit, MI USA; 5 Department of Research & Development Center, John D. Dingell VA Medical Center, Detroit, MI USA

**Keywords:** Ischemia/reperfusion, Postconditioning, Synaptogenesis, Neuroplasticity,, Angiogenesis

## Abstract

There remain debates on neuroprotection and rehabilitation techniques for acute ischemic stroke patients. Therapeutic physical exercise following stroke has shown promise but is challenging to apply clinically. Ischemic conditioning, which has several clinical advantages, is a potential neuroprotective method for stroke rehabilitation that is less understood. In the present study, the rehabilitative properties and mechanisms of physical exercise and remote ischemic postconditioning (RIPostC) after stroke were compared and determined. A total of 248 adult male Sprague-Dawley rats were divided into five groups: (1) sham, (2) stroke, (3) stroke with intense treadmill exercise, (4) stroke with mild treadmill exercise, and (5) stroke with RIPostC. Focal ischemia was evaluated by infarct volume and neurological deficit. Long-term functional outcomes were represented through neurobehavioral function tests: adhesive removal, beam balance, forelimb placing, grid walk, rota-rod, and Morris water maze. To further understand the mechanisms underlying neurorehabilitation and verify the presence thereof, we measured mRNA and protein levels of neuroplasticity factors, synaptic proteins, angiogenesis factors, and regulation molecules, including HIF-1α, BDNF, TrkB, and CREB. The key role of HIF-1α was elucidated by using the inhibitor, YC-1. Both exercise intensities and RIPostC significantly decreased infarct volumes and neurological deficits and outperformed the stroke group in the neurobehavioral function tests. All treatment groups showed significant increases in mRNA and protein expression levels of the target molecules for neurogenesis, synaptogenesis, and angiogenesis, with intermittent further increases in the RIPostC group. HIF-1α inhibition nullified most beneficial effects and indicative molecule expressions, including HIF-1α, BDNF, TrkB, and CREB, in both procedures. RIPostC is equally, or superiorly, effective in inducing neuroprotection and rehabilitation compared to exercise in ischemic rats. HIF-1α likely plays an important role in the efficacy of neuroplasticity conditioning, possibly through HIF-1α/BDNF/TrkB/CREB regulation.

## Introduction

Stroke is the fifth leading cause of death and the leading cause of disability worldwide [1]. Over the past two decades, the economic and disease burden of stroke has increased dramatically and is anticipated to continue growing due to aging populations and shifting dependency ratios [2]. Meanwhile, the development of neuroprotective and rehabilitative strategies remains a challenge in improving the quality of life in post-stroke patients after initial life-saving measures.

The effect of physical exercise in neuroprotection and rehabilitation following ischemic stroke has been extensively studied and is recommended for all post-stroke patients [3]. Physical exercise mitigates many detrimental consequences of stroke, including memory loss [4], neurological impairment [5], and motor function [6]. However, there is significant variability in the extent of neuroprotection conferred by exercise, depending on the time of initiation, the dosage, and the type of activity [7]. While there is ample recent evidence to support the ability of physical exercise to promote recovery through increased neuroplasticity, a key component of successful rehabilitation [8], a number of obstacles still remain in implementing post-stroke exercise in stroke rehabilitation. Stroke patients are generally refractory to physical activity and are likely to face challenges in complying with post-stroke exercise plans, especially in the earlier post-stroke period [9], which can impair rehabilitation potential during the most salient stages of disease progression. Given patients’ varying forms and extent of disability after a stroke, it also remains a challenge to research and implement a standardized treatment plan for stroke patients. Patients’ amenability to physical activity may vary depending on age, motivation, and other factors. Moreover, recent clinical trials of stroke patients undergoing early physical exercise did not show consistent rehabilitative benefits and instead revealed that very early exercise rehabilitation, such as within 24 h of the stroke, may worsen the post-stroke prognosis [10]. In order to bridge these challenges in the continuum of post-stroke rehabilitation and care, there is growing interest in alternative rehabilitation options, namely ischemic conditioning.

Ischemic conditioning leverages the neuroplastic and neuroprotective benefits of temporary and controlled ischemia for therapeutic benefit [11]. A variety of ischemic conditioning methods have been employed, including ischemic preconditioning (IPreC, exposure to moderate hypoxia prior to an ischemic event) and ischemic postconditioning (IPostC, exposure to moderate hypoxia after an ischemic event) [12]. IPostC has been shown to increase cerebral perfusion, prevent neuronal cell death, and improve cognitive function, notably in spatial learning and memory impairment, in ischemic brains [13–16]. Remote ischemic postconditioning (RIPostC) is a conditioning method that induces brief, focal hypoxia in the limbs using blood pressure cuffs [17]. It has been employed in a variety of clinical situations for diverse therapeutic goals [18]. RIPostC may confer neuroprotection through multiple mechanisms, including enhanced cerebral perfusion, formation of cerebral collaterals, and increased tolerance to cerebral ischemia [19, 20]. As a result, it has been shown to improve motor function recovery [21], reduce infarct size [22], and minimize cerebral injury by attenuating apoptosis [23, 24]. Furthermore, it is a passive therapeutic measure for the patient and its use is not dependent on his or her level of motivation or post-stroke disability. As an attractive option to augment or replace post-stroke physical exercise, ischemic conditioning may confer neurorehabilitation to stroke patients with high disease burdens. However, RIPostC is yet to be thoroughly studied in the setting of stroke rehabilitation.

The present study aimed to determine whether RIPostC following ischemic stroke can be an effective alternative or augmentative rehabilitation strategy to physical exercise. In order to comprehensively understand and compare the efficacy of exercise and ischemic conditioning, this study further elucidated the important molecular regulations of neurorehabilitative mechanisms. We measured various proteins and biochemical pathways known to be involved in rehabilitation of the ischemic brain in order to assess the benefit of RIPostC. To assess neuroplasticity, we measured neural microtubule proteins (Tau), growth associated protein 43 (GAP-43) and synaptic proteins (postsynaptic density protein 95 (PSD-95) and synaptophysin (SYN)); for angiogenesis, we measured vascular endothelial growth factor (VEGF), Angiopoietin-1 (Ang-1), and Angiopoietin-2 (Ang-2); and for neuroplasticity regulation, we measured brain-derived neurotrophic factor (BDNF), tropomyosin receptor kinase B (TrkB), cAMP-response-element binding protein (CREB), and nerve growth factor (NGF). We further elucidated the key role of hypoxia-inducible factor 1α (HIF-1α) underlying the rehabilitative mechanism by using the inhibitor, YC-1.

## Materials and Methods

### Animals

**Fig. 1:**
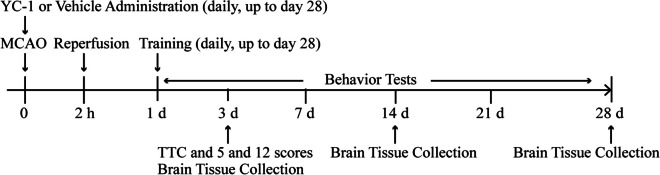
(A) Illustration of the experimental timelines. Rats were subjected to 2-h middle cerebral artery occlusion (MCAO) followed by daily treadmill exercise and conditioning from 1 until 28 days after reperfusion. YC-1 or a vehicle was i.p. injected every day up to day 28

A total of 248 adult male Sprague-Dawley rats (280–300 g, Vital River Laboratory Animal Technology Co., Ltd., Beijing, China) were used in this study. The protocol was approved by the Animal Care and Use Committee of the Capital Medical University, and the study was conducted in accordance with the National Institutes of Health Guide for the Care and Use of Laboratory Animals (USA). The animals were randomly divided into five groups with or without YC-1 administration: (1) sham (*n*=8×3), (2) stroke (*n*=8×7), (3) stroke subjected to intense treadmill exercise (*n*=8×7), (4) stroke subjected to mild treadmill exercise (*n*=8×7), and (5) stroke subjected to non-invasive RIPostC (*n*=8×7). Both exercise and RIPostC protocols were initiated at 24 h of reperfusion and continued to one of the following end points: days 3, 14, and 28 after reperfusion. Ischemic animals were randomly assigned into 7 subgroups: (1–2) histological analysis at day 3 with (1) or without (2) YC-1 administration, (3–7) the tissue collection at day 3 with (3) or without (4) YC-1 administration, at day 14 (5), at day 28 with (6) or without (7) YC-1 administration exercise. The experimental timelines are illustrated in Fig. 1.

### Focal Cerebral Ischemia

This model has been widely used by many researchers, including us [17]. Rats were fasted from food but allowed free access to water for 12–16 h before surgery. Briefly, rats were initially anesthetized in a chamber with 3% isoflurane and a mixture of 70% nitrous oxide and 30% oxygen and maintained with a facemask using 1% isoflurane delivered from a calibrated precision vaporizer. Poly-l-lysine-coated intraluminal nylon (4.0) sutures were used to yield consistent infarcts, greatly reducing inter-animal variability. During the unilateral, 2-h middle cerebral artery occlusion (MCAO) procedure, cerebral blood flow (CBF), blood pCO_2_ and pO_2_, mean arterial pressure, and rectal temperature were monitored continuously. Rectal temperatures were maintained between 36.5 and 37.5 °C using a circulating heating pad and a heating lamp throughout the surgery.

### YC-1 Administration

Rats were treated with 3-(5′-hydroxymethyl-2′-furyl)-1-benzyl indazole (YC-1), an HIF-1α inhibitor (Selleck, Houston, USA dissolved in a solution of 1% dimethyl sulfoxide, DMSO), which is known to downregulate HIF-1α at the translational level. YC-1 inactivates the COOH-terminal transactivation domain (CAD) of HIF-1α and stimulates factor inhibiting HIF (FIH) binding, further suppressing HIF-1α [25]. Five milligrams/kg YC-1 or a vehicle (PBS) was i.p. injected every day up to day 28. [26].

### Treadmill Exercise

Animals were run on a four-lane treadmill (ZS-PT-II, ZS Dichuang Instruments, Inc., Beijing, China) at a constant speed 30 m/min for 30 min each day for intense exercise level. For mild exercise, animals were run at 5 m/min for the first 10 min, 9 m/min for the second 10 min, and 12 m/min for the last 10 min on 1 and 2 days, and then 12 m/min for 30 min on the third and subsequent days, as described previously by us [27]. All animals were housed in groups of three in standard cages for equal times.

### RIPostC

RIPostC was achieved by 3 cycles of ischemia (10 min) and reperfusion (10 min) of both femoral arteries by placing a thin elastic tourniquet around the upper third of the limb in a tight position to occlude the arterial blood supply, as described previously by us [28]. The ischemia of the hind limbs was confirmed by cyanosis and hypothermia of limb within 20 s. Reperfusion was confirmed when the skin color returned to the normal pink color within 20 s.

### Neurological Deficits

The modified scoring systems (5 and 12 scores) proposed by Zea Longa (5 scores) and Belayev et al. (12 scores) were used to examine the severity of neurological deficits in rats before and after 24 h of reperfusion [29]. The severity and consistency of brain damage within each group was highly important in this study. After MCAO, the modified scoring systems for neurological deficits were used to confirm brain injury. After MCAO, rats with scores of 2 or below or dead were considered to represent the unsuccessful establishment of the MCAO model and were consequently excluded (about 10%); exclusion was then confirmed on autopsy by lack of an ischemic core, indicating an unsuccessful surgery.

### Cerebral Infarct Volume

After 3 days of reperfusion in rats with 2 h MCAO, the brains were resected from ischemic rats and cut into 2-mm-thick slices (brain matrix) and treated with 2,3,5-triphenyltetrazolium chloride (TTC; Sigma-Aldrich, St. Louis, MO, USA) for staining [30]. An indirect method for calculating infarct volume was used to minimize error caused by edema.

### Neurobehavioral Functions

The functional outcomes were determined by a series of tests, including adhesive removal, beam balance, forelimb placing, grid walk, and rota-rod (R03-1; Xin-Ruan Instruments, Inc., Shanghai, China) at days 1, 3, 7, 14, 21, and 28, and the Morris water maze (ZS-II; ZS Dichuang Instruments, Inc., Beijing, China) at 23 through 27 days after MCAO as described previously by us [31].

In the adhesive removal test, an adhesive tape was attached on the palmar surface of the forepaw. The times taken for the first attempt to touch the tape and to remove the tape were recorded and analyzed. In the beam balance test, rats were placed on a narrow wooden beam (122×2.5×42 cm). Motor function was scored from 0 to 6 as previously described [31] for their performance (0=no attempts to stay on the beam; 1=attempts to stay on the beam without movement; 2=attempts to cross the beam but fails; 3=crosses the beam but the contralateral hindlimb slips >50%; 4 = crosses the beam but the contralateral hindlimb slips <50%; 5= crosses the beam but the contralateral hindlimb slips once; 6=crosses the beam without a slip). In the forelimb-placing test, rats were held gently with forelimbs close to the tabletop, and lightly brushed the tabletop with each side of their vibrissa. The ability of rats to place the preferred forelimb on the edge of the table was recorded 10 times, and placing rates were calculated. In the grid walk test, rats were placed on a wire grid (100×25×50 cm) and allowed to freely walk from one end to the other. The total number of foot slips, when the forepaw failed to accurately hold onto the rung, was recorded. In the rota-rod test, rats were placed on a rotating drum and forced to run with speeds accelerating from 4 to 40 rpm within 200 s. The duration that the animals stayed on the rotating rod was recorded. In the Morris water maze, the rats were placed into the pool (diameter 150 cm) in one of the four starting locations and allowed to swim for 120 s to find the hidden platform (diameter 10 cm). Latencies to find the hidden platform, time spent on each quadrant, and swim speeds were recorded.

### mRNA Expression by Real-Time PCR

At 3, 14, and 28 days following exercise or RIPostC regimens, isolated cerebral samples were homogenized and RNA was isolated using the Trizol reagent (Invitrogen, Carlsbad, CA) as described previously by us [32]. Total RNA was then converted into cDNA using the High Capacity cDNA Reverse Transcription Kit (Applied Biosystems, Foster City, CA). The quantification of gene expression was determined by Prism 7500 real-time PCR (Applied Biosystems, CA, USA). All reactions were performed under the following conditions: 95 °C for 15 minutes, 40 cycles of 95 °C for 10 s, and 60 °C for 30 s. β-Actin was used as the control gene and all data are represented as relative mRNA expression on gene expression. The primers for rat BDNF, NGF, PSD-95, SYN, Tau, GAP43, VEGF, Ang-1, Ang-2, TrkB, CREB, HIF 1α, and β-actin are shown in Table 1.

**Table 1:** Primer sequences of each target gene

Target gene	Forward	Reverse
BDNF	5′-GAGCGTGTGTGACAGTATTAG-3′	5′-GTAGTTCGGCATTGCGAGTTC-3′
NGF	5′-AGCGTAATGTCCATGTTGTTCTAC -3′	5′-TGCTATCTGTGTACGGTTCTGC -3′
PSD95	5′-TGCACTATGCTCGTCCCATCATCA-3′	5′-TGTGCCTGGATGTCCTTCTCCATT-3′
SYN	5′-AAGGCCTGTCCGATGTGAAG-3′	5′-AGGAAGCCAAACACCACTGA-3′
Tau	5′-ACCATGGCTTAAAAGCTGAAGAAG-3′	5′-CGGCCACTCGAGCTTGAGTC-3′
GAP43	5′-GATGCGGCCCCTTCAGAG-3′	5′-CCTTGGCTGGGCCATCTT-3′
VEGF	5′-AGAAAGCCCATGAAGTGGTG-3′	5′-ACTCCAGGGCTTCATCATTG-3′
Ang-1	5′-ACATGGGCAATGTGCCTACA-3′	5′-TTCTCAAGTTTTTGCAGCCACTG-3′
Ang-2	5′-AGAGTACAAAGAGGGCTTCGG-3′	5′-ATACAGAGAGTGTGCCTCGC-3′
TrkB	5′-GTGGAGGAAGGGAAGTCTGTG-3′	5′-CAGTGGTGGTCTGAGGTTGGA-3′
CREB	5′-TACCCAGGGAGGAGCAATACA-3′	5′-GGTGCTGTGCGAATCTGGTAT-3′
HIF 1α	5′-TCAAGTCAGCAACGTGGAAG-3′	5′-TATCGAGGCTGTGTCGACTG-3′
β-Actin	5′-TCATGAAGTGTGACGTTGACATCCGT-3′	5′-CCTAGAAGCATTTGCGGTGCAGGATG-3′

### Protein Expression by Western Blot

At the same time points following the procedures [32], proteins were extracted from the MCA-supplied brain regions and loaded onto SDS-polyacrylamide gel for electrophoresis. Gel transfer to a PVDF membrane was performed under 200 V for 1 h. Membranes were blocked with 5% skimmed milk. Membranes were incubated with primary antibodies (1:1000, rabbit anti-BDNF, rabbit anti-NGF, rabbit anti-PSD-95, rabbit anti-SYN, mouse anti-Tau, rabbit anti-GAP43, rabbit anti-VEGF, rabbit anti-Ang-1, rabbit anti-Ang-2, rabbit anti-TrkB and rabbit anti-CREB, Abcam, MA, USA; 1:500, rabbit anti-HIF-1α, Santa Cruz Biotechnology, Inc. CA, USA) for 24 h at 4 °C. The secondary antibody was goat anti-rabbit IgG-HRP (Santa Cruz), which was incubated for 1 h at room temperature for all primary antibodies. Western blot images for each of the antibodies were analyzed using an image analysis program (ImageJ 1.42, National Institutes of Health, Bethesda, MD, USA) to quantify protein expression in terms of relative image density.

### Statistical Analysis

Statistical analyses were performed with SPSS Statistics for Windows, Version 17.0 (SPSS Inc., Chicago, IL, USA). Differences among groups were assessed using one-way ANOVA with a significance level of *P*<0.05. Post hoc comparison among groups was performed using the least significant difference method.

## Results

### Brain Damage

**Fig. 2:**
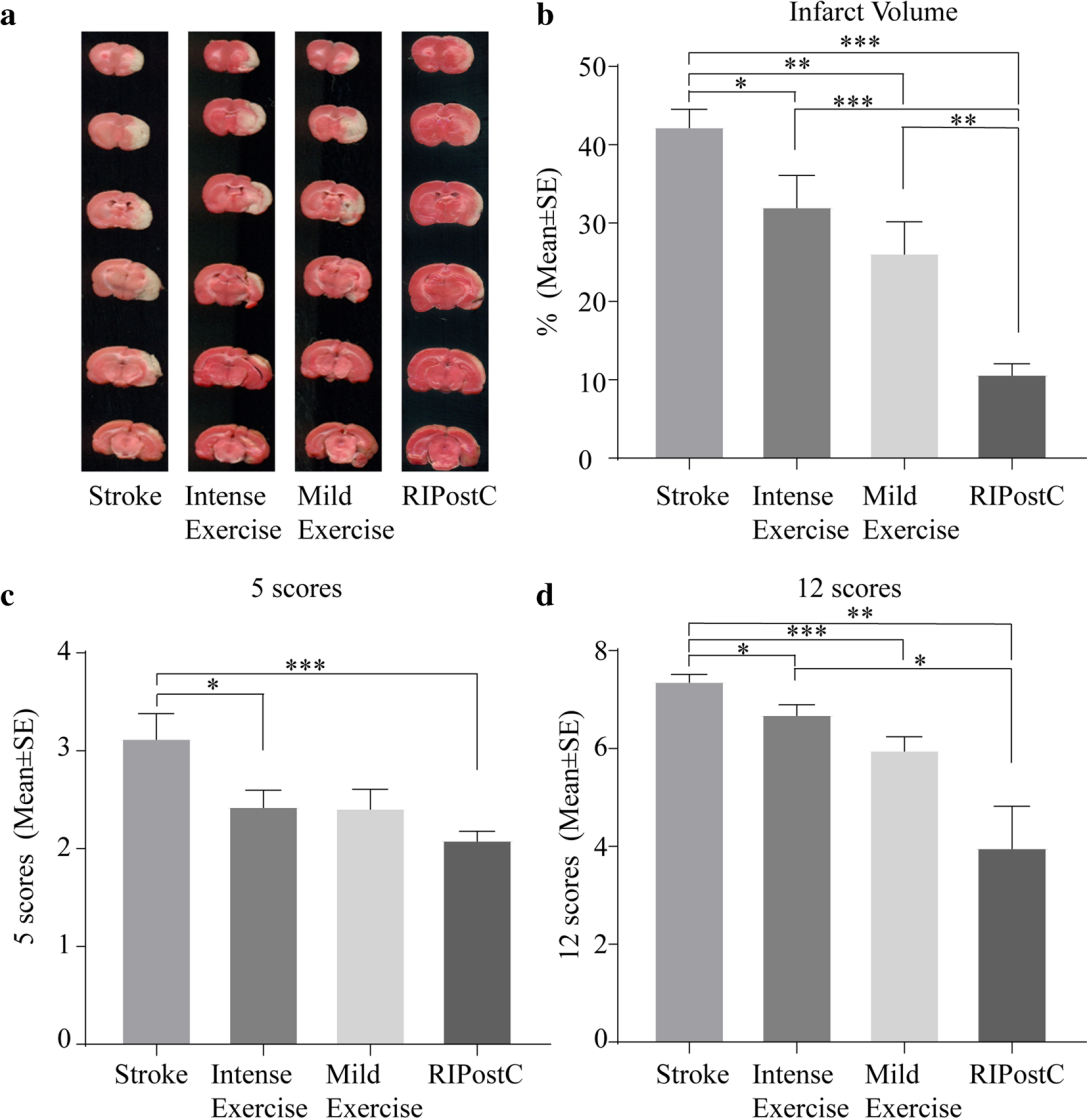
Exercise and RIPostC reduced brain infarct. **a** Infarct volume reduction in the penumbra region of ischemic territory supplied by MCA. **b** Intense exercise, mild exercise, and RIPostC significantly decreased infarct volumes as compared to stroke rats without treatment. Further decreases were seen with RIPostC. **c**, **d** Intense exercise, mild exercise, and RIPostC significantly reduced neurological deficits as compared to stroke rats without treatment. Further reduction was seen with RIPostC as well. The data was presented as the mean ± SEM, *n*=8. *p<0.05, **p<0.01, ***p<0.001

A large infarct was seen after 2 h MCAO followed by 3-day reperfusion (42.5%). Both exercise (intense and mild) and RIPostC significantly decreased the infarct volumes to 32.3%, 26.3%, and 10.9%, respectively. RIPostC had a significantly additional decrease in infarct volume compared to the two exercise groups (Fig. 2a, b).

Neurological deficits were detected by the 5 or 12 scoring systems after stroke (Fig. 2c, d). Deficits were decreased significantly in all groups, while RIPostC induced an additionally significant decrease in the 12 scoring systems compared to the intense exercise group (Fig. 2d).

### Functional Outcome

**Fig. 3:**
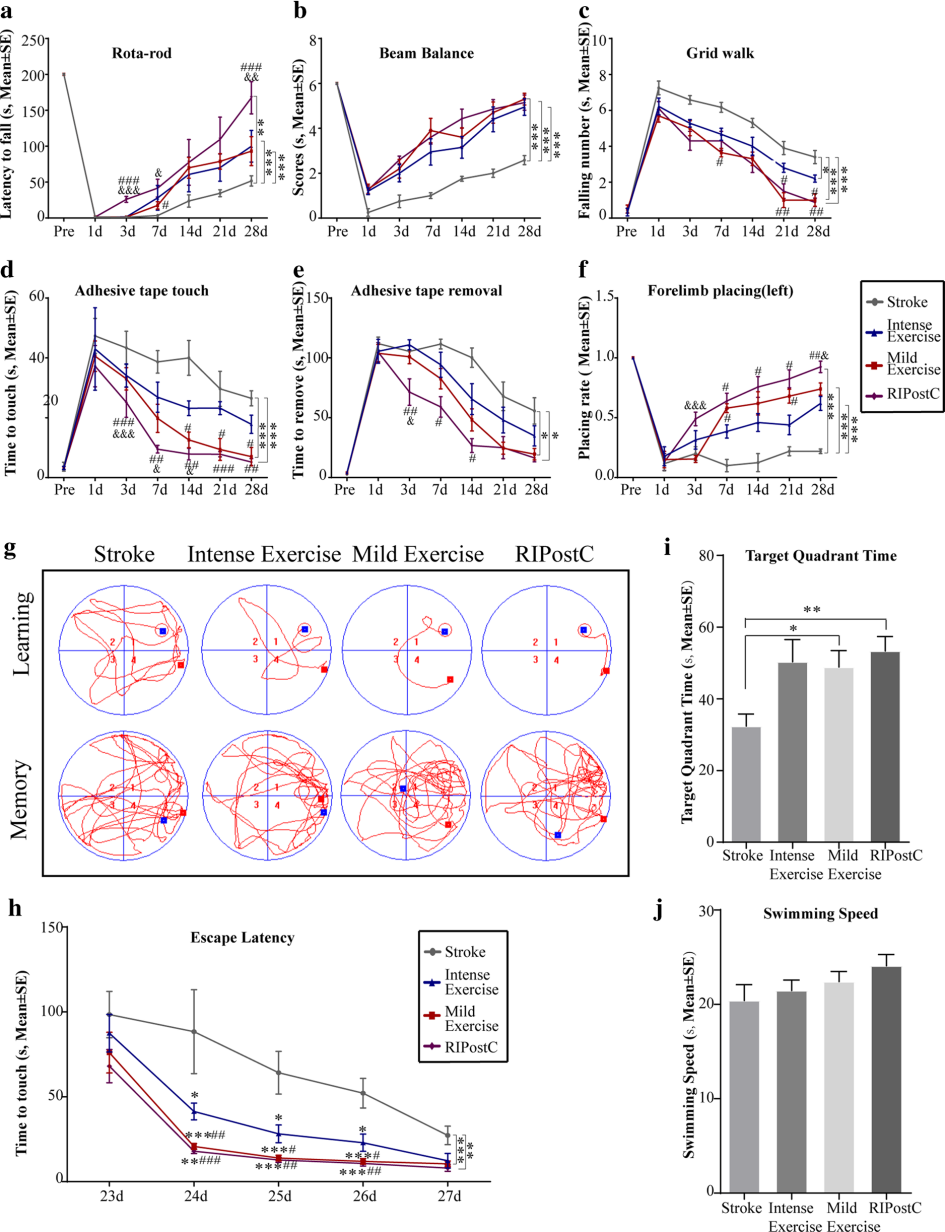
Exercise and RIPostC improved neurobehavioral function. (A–F) Neurobehavioral functional outcomes were significantly improved after intense exercise, mild exercise, and RIPostC as compared to stroke rats without treatment. Further improved performance was seen with RIPostC. (G–J) Learning and memory ability was examined by the Morris water maze test. (G) Representative images of swimming paths during learning at 27 days and memory at 28 days after MCAO. (H) Latency to locate the submerged platform was significantly shortened after intense exercise, mild exercise, and RIPostC. (I) The rats wasted less time in the other quadrants and spent more time in the target quadrant with the hidden platform after mild exercise and RIPostC. (J) Swimming speeds were not improved significantly with exercise and RIPostC, indicating similar gross motor skills. The data was presented as the mean ± SEM, *n*=8. *p<0.05, **p<0.01, ***p<0.001 vs stroke; ^#^*p*<0.05, ^##^*p*<0.01, ^###^*p*<0.001 vs intense exercise; ^&^*p*<0.05, ^&&^*p*<0.01, ^&&&^*p*<0.001 vs mild exercise

The latency to fall off the rota-rod was significantly increased by both exercise protocols and RIPostC as compared to the stroke-only rats at 3 through 28 days (Fig. 3(A)). Performances of the RIPostC rats were further improved significantly on 3, 7, and 28 days. Similar trends in the functional outcomes were observed in all other tests: the beam balance, grid walk, adhesive removal, and forelimb placing tests (Fig. 3(B–F)).

We also evaluated cognitive deficits using the Morris water maze at 23 through 27 days (Fig. 3(G–J)). Both exercise regimens and RIPostC significantly shortened the latency to locate the hidden platform as compared to the stroke-only rats (Fig. 3(G–H)). Compared to the stroke rats, mild exercise and RIPostC rats spent less time in other quadrants and spent more time in the target quadrant with the hidden platform (Fig. 3(I)). There were no significant differences in swimming speeds between the groups, suggesting similar gross motor skills (Fig. 3(J)).

### Neuroplasticity, Synaptogenesis, and Angiogenesis

**Fig. 4:**
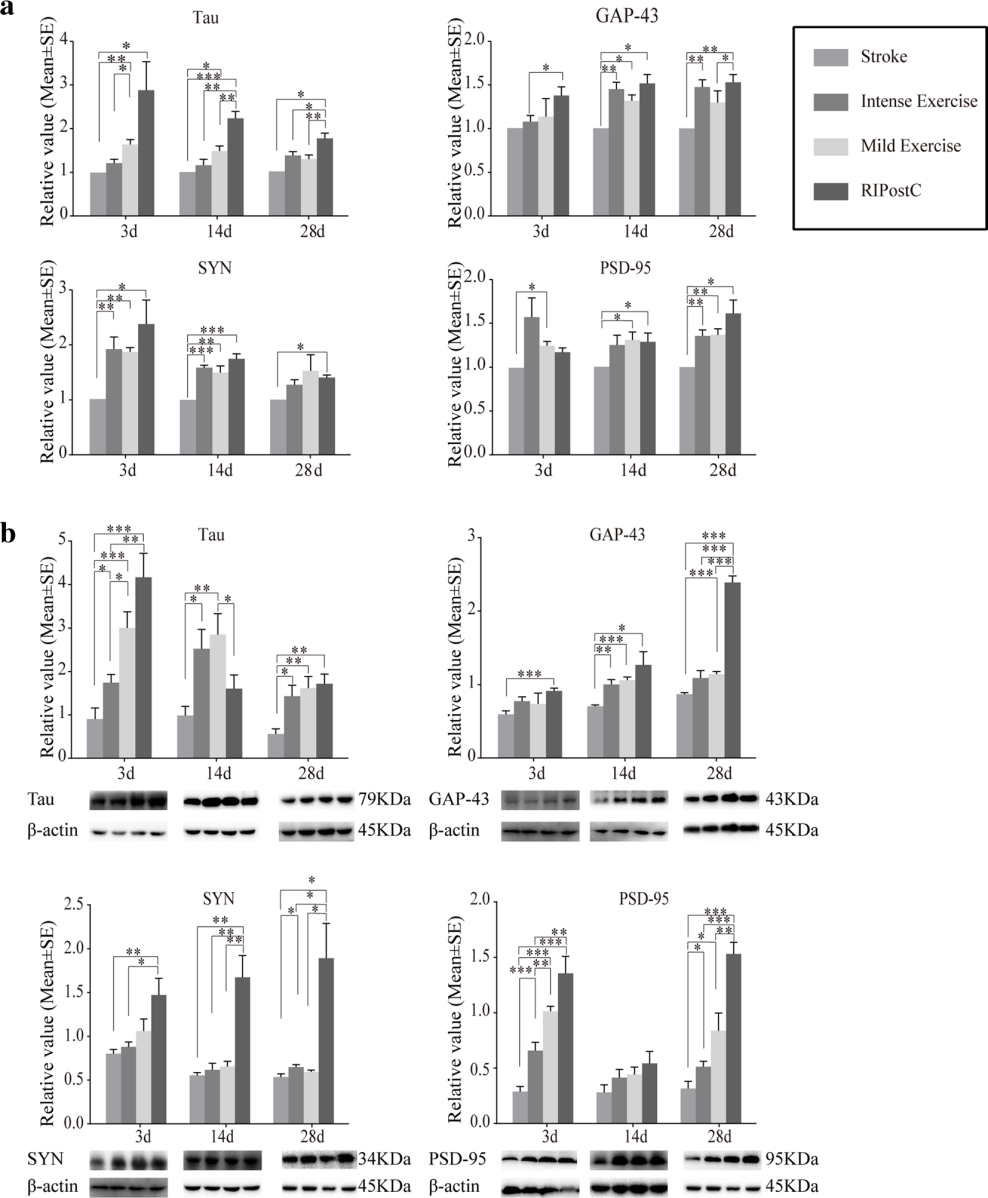
Exercise and RIPostC enhanced neuroplasticity and synaptogenesis (Tau, GAP-43, SYN, PSD-95), shown by mRNA and protein expressions. (A) mRNA expressions of Tau, GAP-43, SYN, and PSD-95 were significantly increased after exercise and RIPostC as compared to stroke rats (Reference at 1). Further increases were seen with RIPostC in Tau and GAP-43. (B) Protein measures of Tau, GAP-43, SYN, and PSD-95 were also significantly increased after exercise and RIPostC in ischemic rats. Further increases were induced by RIPostC in Tau, GAP-43, SYN, and PSD-95. The data was presented as the mean ± SEM, *n*=8. *p<0.05, **p<0.01, ***p<0.001

As compared to the stroke-only rats, both exercise regimes and RIPostC yielded significantly increased mRNA and protein expressions of Tau, GAP-43, SYN, and PSD-95 (Fig. 4(A, B)) at various time points from 3 through 28 days. In comparison to the exercise groups, RIPostC further significantly increased mRNA and protein expressions of the target molecules at various time points. Together, these results indicate a significant role of both exercise levels and RIPostC protocols on neuroplasticity after ischemic stroke, with greater influence of RIPostC on neuroplasticity.

**Fig. 5:**
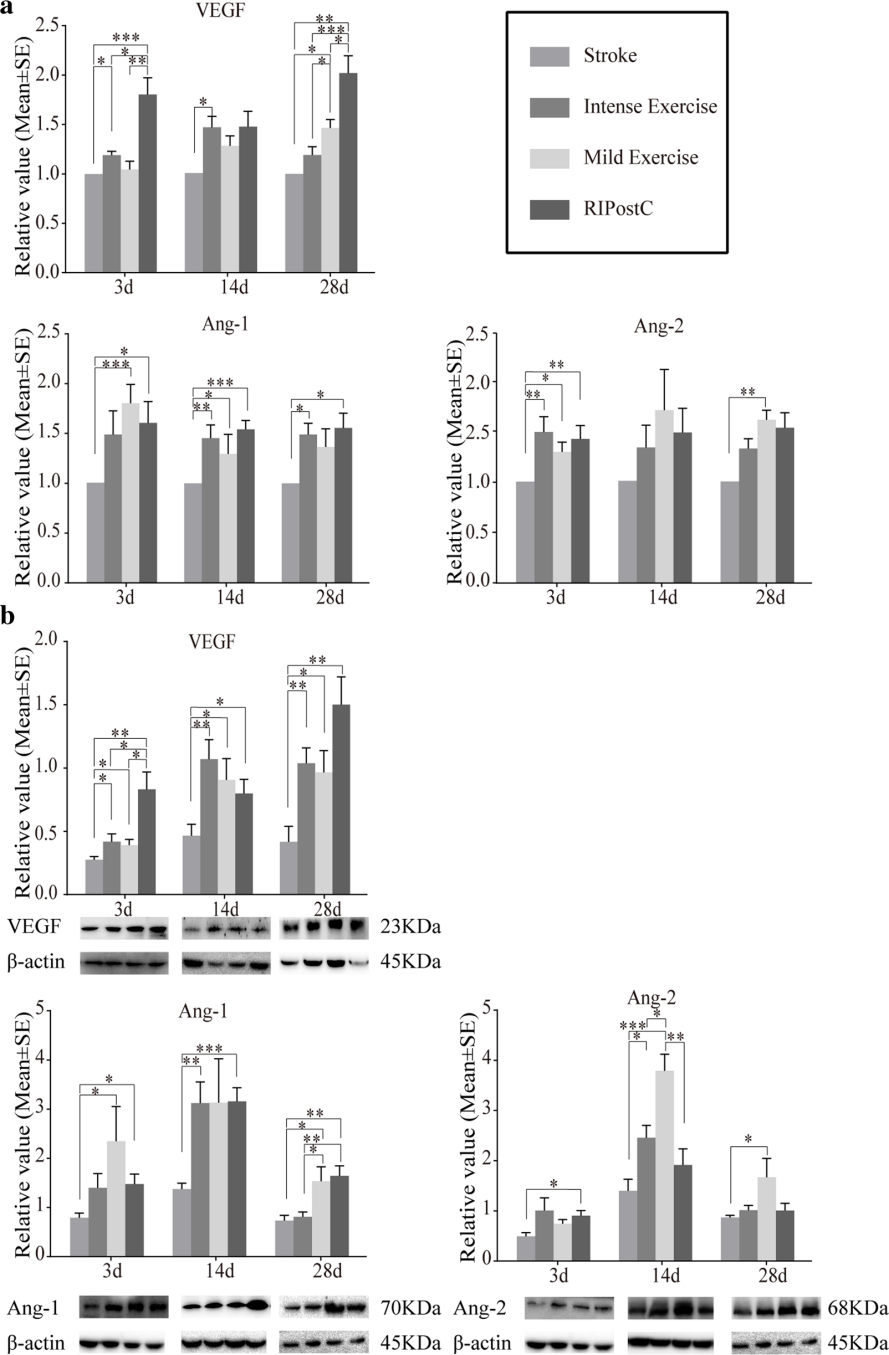
Exercise and RIPostC enhanced angiogenesis, shown by mRNA and protein expressions of VEGF, Ang-1, and Ang-2. (A) mRNA expressions of VEGF, Ang-1, and Ang-2 were significantly increased after exercise and RIPostC as compared to stroke rats (Reference at 1), with further increasing with RIPostC in VEGF. (B) Protein measures of VEGF, Ang-1, and Ang-2 were significantly increased by exercise and RIPostC, in comparison to ischemic rats. Further increases were induced by RIPostC in VEGF and Ang-1. The data was presented as the mean ± SEM, *n*=8. *p<0.05, **p<0.01, ***p<0.001

Both exercise protocols and RIPostC significantly increased angiogenic factors, including VEGF, Ang-1, and Ang-2 as compared to the stroke-only group (Fig. 5). The expression of VEGF mRNA was further significantly increased by RIPostC compared to the two exercise groups (Fig. 5(A)). VEGF protein expression was also enhanced by RIPostC on day 3 (Fig. 5(B)). Ang-1 protein expression was further increased significantly at day 28 by mild exercise and RIPostC compared to the intense exercise group, while Ang-2 protein expression was significantly enhanced at day 14 by mild exercise.

### Regulation Signaling on Neural Plasticity

**Fig. 6:**
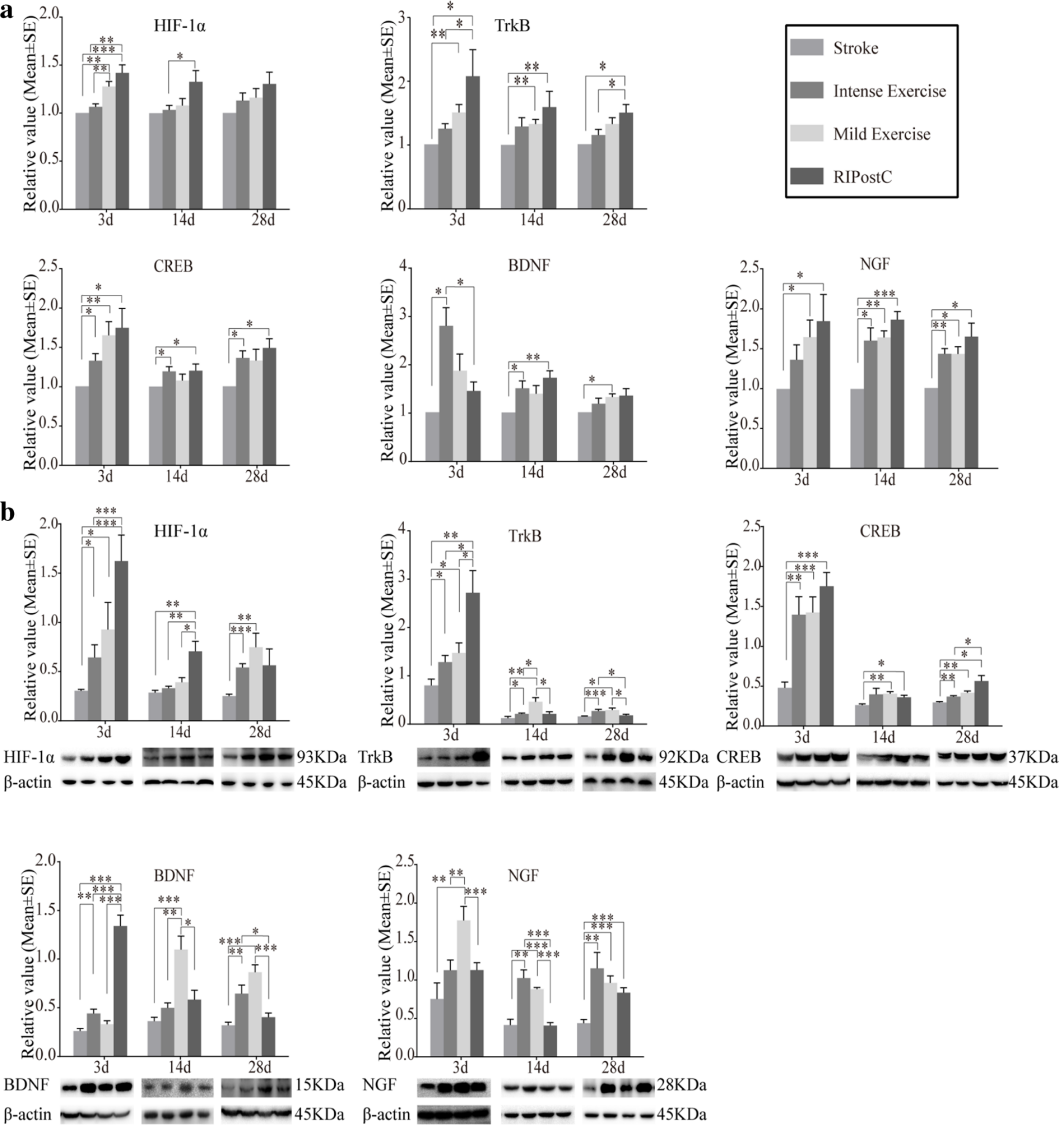
Exercise and RIPostC enhanced neural plasticity regulation, shown by mRNA and protein expressions of HIF-1α, TrkB, CREB, BDNF, and NGF. (A) mRNA expressions of HIF-1α, TrkB, CREB, BDNF, and NGF were increased significantly by exercise and RIPostC in ischemic rats (Reference at 1). HIF-1α and TrkB were further increased with RIPostC. (B) Protein measures of HIF-1α, TrkB, CREB, BDNF, and NGF were also significantly increased after exercise and RIPostC. HIF-1α, TrkB, CREB, and BDNF were further increased with RIPostC. The data was presented as the mean ± SEM, *n*=8. *p<0.05, **p<0.01, ***p<0.001

The mRNA and protein expressions of HIF-1α, TrkB, CREB, BDNF, and NGF were assessed (Fig. 6). At 3 through 28 days after stroke, expression of mRNA but not protein of all the above molecules in the stroke group was observed at normal levels as compared to the sham control (data not shown). Both exercise and RIPostC significantly increased HIF-1α, TrkB, CREB, BDNF, and NGF expressions at both mRNA (Fig. 6(A)) and protein (Fig. 6(B)) levels. Specifically, the intense exercise protocol showed a significant increase in the mRNA expressions of HIF-1α, TrkB, CREB, BDNF, and NGF at different time points as compared to the stroke groups. RIPostC significantly further increased mRNA levels of HIF-1α and TrkB. The two exercise protocols and RIPostC protocol significantly increased protein levels of HIF-1α, TrkB, CREB, BDNF, and NGF (Fig. 6(B)). RIPostC, again, further increased significantly the protein levels of HIF-1α, TrkB, CREB, and BDNF.

### HIF-1α Inhibition Aggravated Brain Infarction

**Fig. 7:**
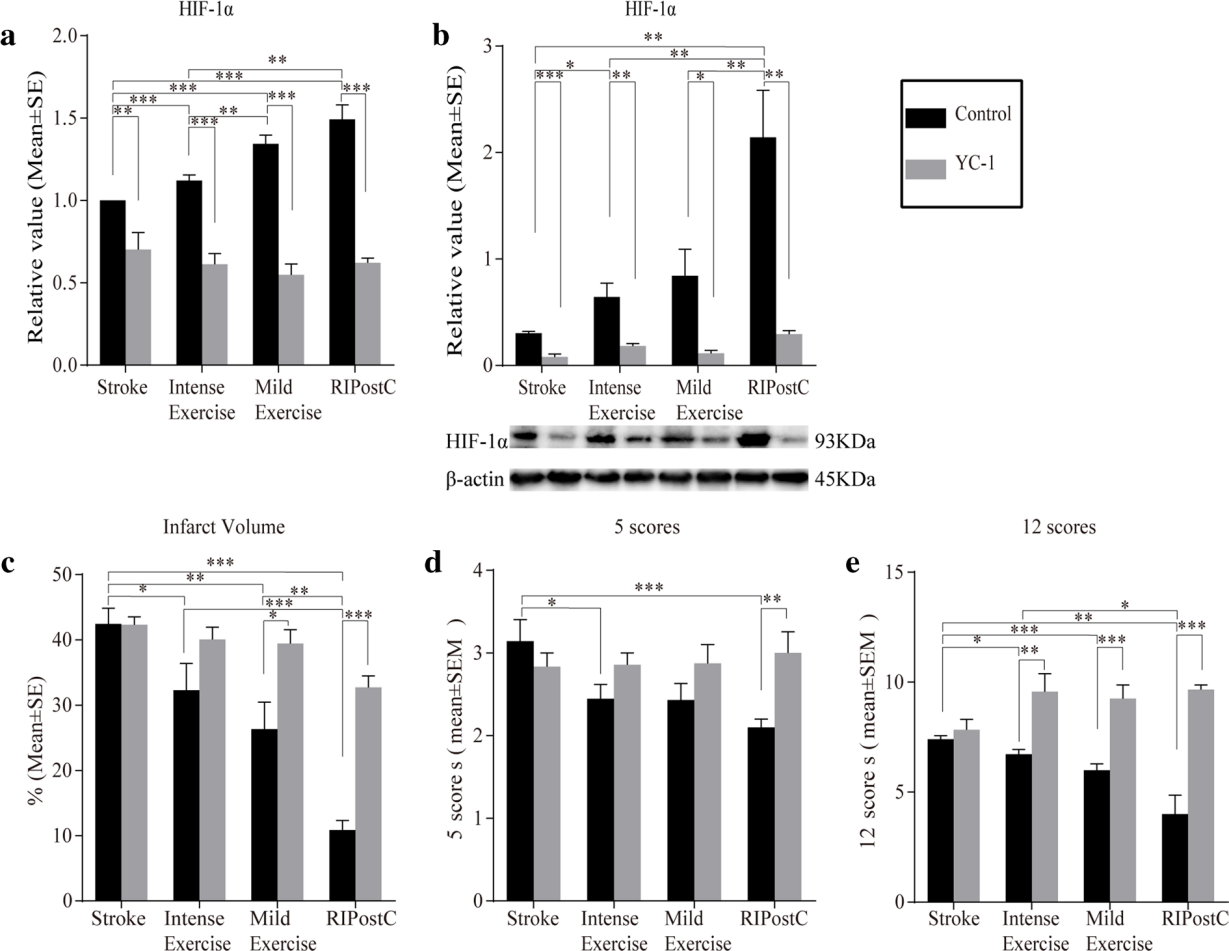
HIF-1α inhibitor, YC-1, significantly abolished brain infarction reduction by exercise and RIPostC. (A) Increases in mRNA expression of HIF-1α by both exercise and ischemic conditioning groups were significantly abolished by YC-1. (B) Increased protein level of HIF-1α in the treatment groups were significantly abolished by YC-1. (C) YC-1 significantly abolished brain infarction reduction by exercise and RIPostC. (D, E) Neurological deficit reduction by exercise and ischemic conditioning were reversed by YC-1. The data was presented as the mean ± SEM, *n*=8. *p<0.05, **p<0.01, ***p<0.001

YC-1, the HIF-1α inhibitor, essentially annulled the increases in mRNA and protein expressions of HIF-1α in all treatment groups at 3 days (Fig. 7(A, B)). The infarct volumes in the stroke group, with or without YC-1, were large and similar at 3 days. As predicted, YC-1 prevented infarct volume reduction in both exercise and ischemic conditioning protocols, partially reversing neuroprotection in all 3 groups (Fig. 7(C)). The high neurological deficits in the stroke group without exercise or ischemic conditioning, detected by the 5 or 12 scoring systems, were not affected by YC-1 administration at 3 days. Again, YC-1 obliterated the neurological deficit improvements by either exercise or ischemic conditioning (Fig. 7(D, E)). This suggests that at relatively later stages of ischemic stroke, HIF-1α is not a key player of brain injury, but of rehabilitative strategies.

### HIF-1α Inhibition Attenuated Long-Term Functional Outcome

**Fig. 8:**
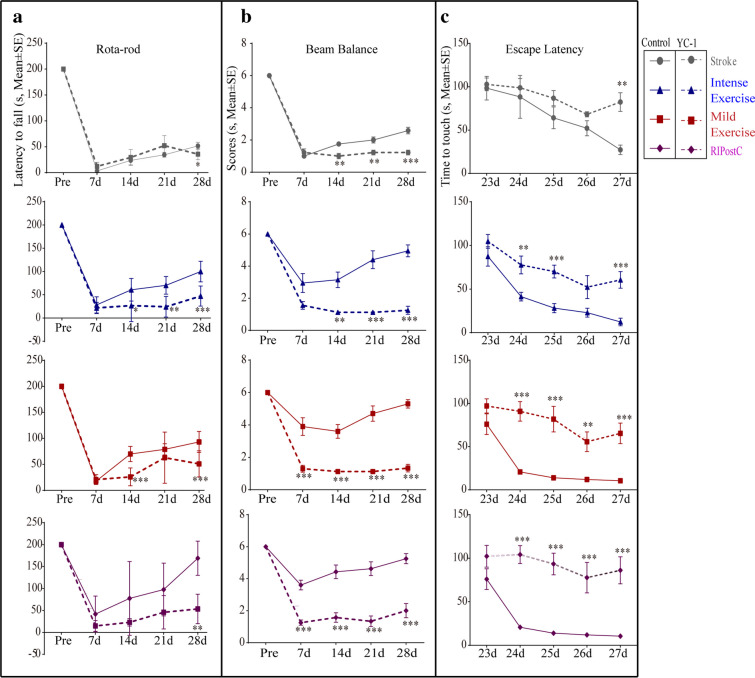
HIF-1α inhibitor, YC-1, significantly attenuated improvements in functional improvements induced by exercise and RIPostC. (A) Neurobehavioral and cognitive functional outcome improvements by exercise and conditioing were significantly reversed by YC-1. The data was presented as the mean ± SEM, *n*=8. *p<0.05, **p<0.01, ***p<0.001

Sensorimotor functions were evaluated by the rota-rod and beam balance tests. The improved motor function after stroke by exercise and ischemic conditioning was significantly decreased by HIF-1α inhibition with YC-1 at 7 through 28 days (Fig. 8(A, B)). We also evaluated cognitive deficits using the Morris water maze at 23–27 days. YC-1 prevented functional improvements in ischemic rats after exercise and ischemic conditioning, displayed by the longer latency to locate the hidden platform as compared to the treatment groups without HIF-1α inhibition (Fig. 8(C)). These results demonstrate the key role of HIF-1α in long-term recovery of sensorimotor functions and spatial learning capabilities by rehabilitative strategies after ischemic stroke.

### HIF-1α Inhibition Abolished Neural Plasticity, Synaptogenesis, And Angiogenesis

**Fig. 9:**
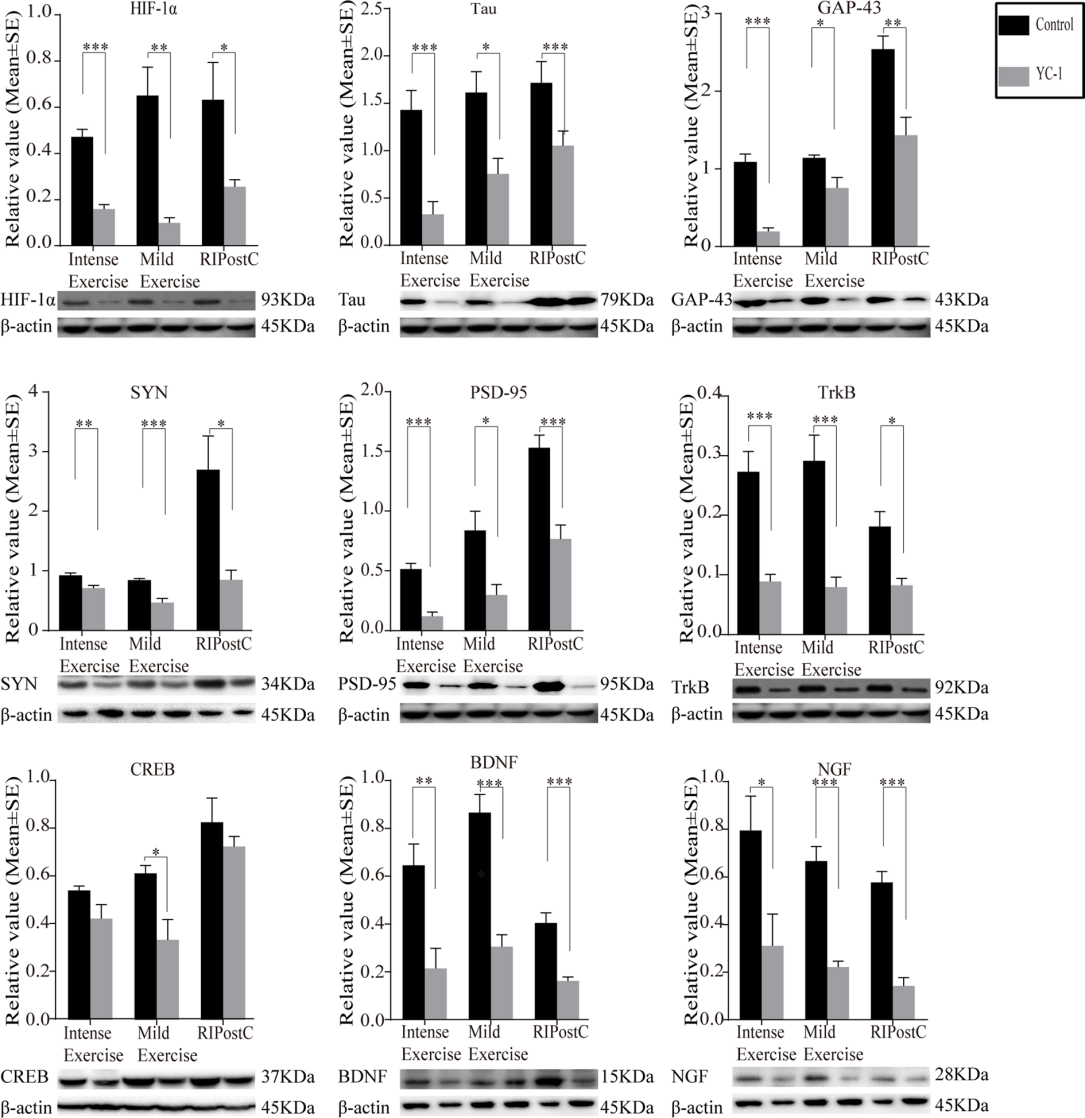
HIF-1α inhibition with YC-1 significantly diminished the promotion of neuroplasticity, synaptogenesis, and neural plasticity regulation by exercise and RIPostC. Increases in protein expression of HIF-1α, Tau, GAP-43, SYN, PSD-95, TrkB, CREB, BDNF, and NGF were all significantly reversed by HIF-1α inhibition with YC-1. The data was presented as the mean ± SEM, *n*=8. *p<0.05, **p<0.01, ***p<0.001

**Fig. 10:**
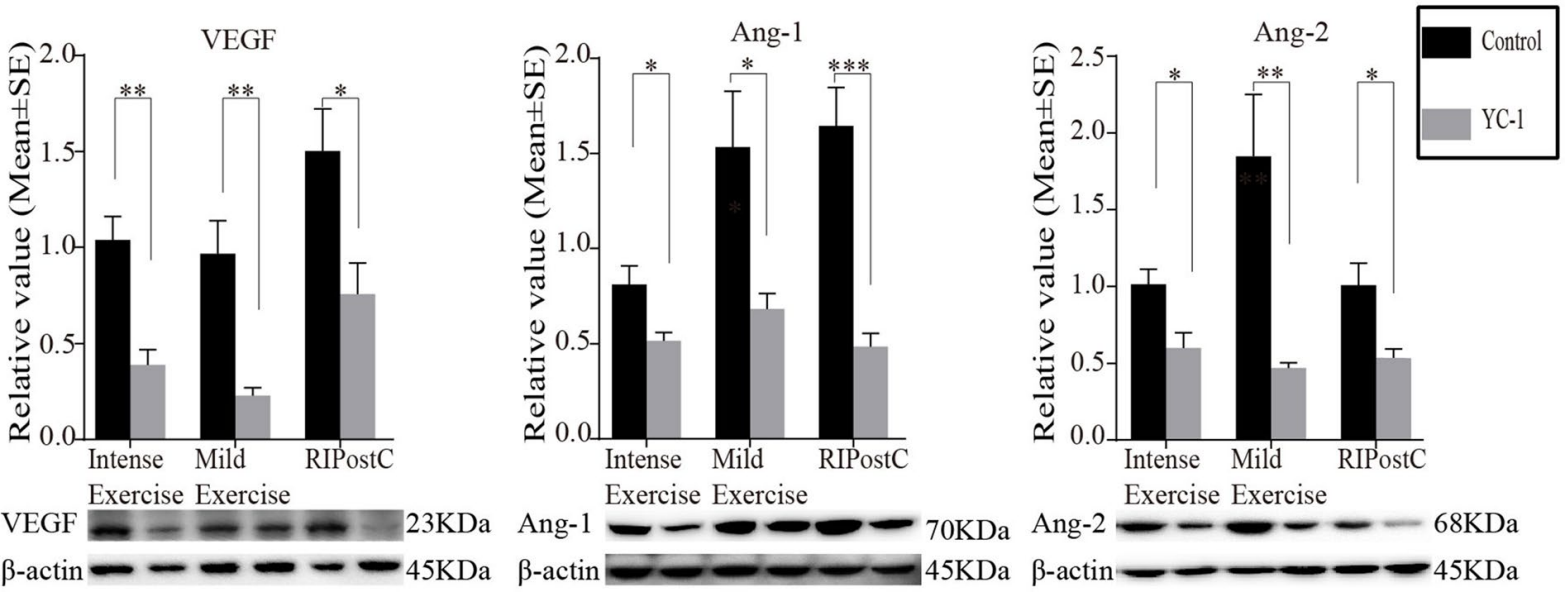
YC-1 reversed the promotion of angiogenesis by exercise and RIPostC. Protein expression of VEGF, Ang-1, and Ang-2 were reduced by YC-1, as compared to those without HIF-1α inhibition after exercise and ischemic conditioning. The data was presented as the mean ± SEM, *n*=8. *p<0.05, **p<0.01, ***p<0.001

YC-1 significantly obliterated protein expression increases of HIF-1α after exercise and ischemic conditioning at 28 days (Fig. 9). Likewise, YC-1 obliterated protein expression increases of Tau, GAP-43, SYN, PSD-95, TrkB, CREB, BDNF, and NGF by exercise regimens and RIPostC (Fig. 9). VEGF, Ang-1, and Ang-2 protein level increases in exercise and RIPostC groups were also abolished by YC-1 (Fig. 10). Together, these results indicate that HIF-1α played a significant role in neuroplasticity, synaptogenesis, and angiogenesis induced by both exercise and RIPostC rehabilitation protocols.

## Discussion

To our knowledge, the present study is the first to directly compare the efficacy of post-stroke exercise and remote ischemic conditioning (RIC) in the context of stroke rehabilitation. It was found that both mild and intense physical exercise and remote ischemic conditioning were rehabilitative following ischemic stroke, as evidenced by improved functional and neurological outcomes as well as reduced infarct volumes. Results from functional and neurological tests particularly demonstrated the efficacy of conditioning in recovering long-term sensorimotor functions and spatial learning capabilities. We further report that neuroplasticity (Tau, GAP-43), synaptogenesis (SYN, PSD-95), angiogenesis (VEGF, Ang-1, Ang-2), and neural plasticity regulation (TrkB, CREB, BDNF, NGF) were associated with improved neurorehabilitation by both exercise and ischemic conditioning after stroke, with further intermittent increases with ischemic conditioning. These observed changes may be regulated by HIF-1α signaling.

### Post-stroke Conditioning on Stroke Rehabilitation

Despite promising results in experimental animal models and one small randomized clinical trial [33], the application of exercise in post-stroke rehabilitation in human populations remains in its infancy and is yet to be entirely established. Recent clinical trials reported a lack of efficacy in improving motor control [6], reduced odds of favorable outcomes [34], and a lack of impact on cognitive function after 3 months [10]. While there may still be a role for early post-stroke exercise in rehabilitation, the benefits seen in previous rodent models have not translated effectively to human clinical trials. Other practical and effective methods should be explored.

Ischemic postconditioning (IPostC) is likely to be relatively simple, non-invasive, and cost-effective to implement in clinical practice. Hypobaric and normobaric hypoxic postconditioning techniques have been developed [35, 36]. Several studies have demonstrated their ability to improve outcomes following global cerebral ischemia [37]. The concept of remote ischemic post conditioning (RIPostC) was first applied in cardiology to reduce myocardial injury following ischemia, with its cardioprotective benefits well established in large-animal models and human trials [38]. Since then, it has been applied to yield diverse benefits in rehabilitation from various ischemic pathologies. A recent clinical trial demonstrated that RIPostC improved cognition in patients with subcortical ischemic vascular dementia [39]. Another study reported that multiple cycles of RIPostC enhanced motor performance after stroke in rodent models [21]. The present study indicated that RIPostC is an auspicious approach for development of novel, effective stroke rehabilitative therapy as compared to exercise strategy. As such, it may become a core rehabilitative measure for victims of cerebral infarcts.

### Similarities in Molecular Pathways Between the Two Rehabilitation Regimes

In order to establish post-stroke ischemic conditioning as a rehabilitative strategy, the present study compared the beneficial effects of ischemic conditioning and exercise on various key molecules. In the present study, functional improvements were observed concurrent to increases in key molecular markers under both exercise and ischemic conditioning, suggesting a convergent neurorehabilitative pathway between post-stroke physical exercise and RIPostC.

GAP-43 is an indicator of neuronal damage and synaptogenesis and has been implicated in axonal sprouting [40]. Currently, there is a paucity of studies regarding the impact of RIPostC on GAP-43 expression. Here, we report that GAP-43 protein levels were increased with RIPostC. This finding is in agreement with an earlier study regarding traumatic brain injury, which indicated that increases in GAP-43 may improve functional outcomes, as evidenced by performances on beam walking and limb foot fault testing [41].

PSD-95 is a scaffolding protein that stabilizes synaptic signaling [42]. SYN is another marker of synaptic plasticity that is implicated in the setting of cerebral ischemia and damage [43]. In this study, RIPostC served as a potential mediator of synaptic reorganization and neuroplasticity through increased expressions of PSD-95 and SYN following ischemic stroke. Consistent with a previous study, the present study found that limb ischemia postconditioning may enhance rehabilitation through SYN induction, leading to increased microtubular stability and subsequent synaptic plasticity and signaling [41].

Ang-1 acts as a vasculature protector [44] and Ang-2 as an angiogenic and pro-inflammatory agent [45]. VEGF is a ubiquitous inducer of angiogenesis and vasculogenesis. The present study demonstrated that VEGF expression was effectively elevated by RIPostC, indicating that RIPostC enhances essential processes of angiogenesis after an ischemic event. This finding aligns with those of a previous study, which observed VEGF upregulation by ischemic conditioning, possibly through downstream effects of HIF-1α, which increases cerebral vascular growth and accelerates cognitive rehabilitation [46].

Studies have demonstrated that BDNF, TrkB, and CREB activate and regulate one another in promoting neuronal survival and protection. BDNF is an indicator of neuronal survival and synaptic plasticity, where it works with TrkB receptors in the brain to improve motor function [47, 48]. They also trigger an intracellular signaling cascade to mediate neuronal survival and differentiation [49], angiogenesis [50], and neural plasticity [51]. The effects of this signaling cascade involve upregulation of neuron protective molecules such as Bcl-2, anti-oxidants, and CREB. Bcl-2, in turn, is involved in attenuation of neuronal apoptosis [38, 52]. CREB plays a role in neurogenesis [53], induces functional recovery, and increases circuit plasticity following stroke [54] by activating anti-oxidants and anti-apoptotic proteins [38]. This pathway provides positive feedback to upregulate BDNF expression [55], which explains the observed coordinated growth of CREB, BDNF, and TrkB by previous studies [56]. The present study demonstrated that RIPostC conferred increases in the levels of these vital angiogenic and neuroplastic compounds that were greater than those seen in physical exercise. This study as well as prior evidence support the notion that hypobaric hypoxia attenuates reperfusion injury through CREB and BDNF regulation [57].

### Reduction of Infarct Volumes

RIPostC was shown to reduce infarct volumes to a greater extent than physical therapy in the ischemic brain. This finding is in accordance with mechanisms discussed in previous studies. RIPostC has been shown to upregulate endogenous tissue kallikrein (TK) levels in circulating blood and local ischemic brain regions [21]. Others have indicated that RIPostC inhibits Bcl-2 interacting-domain death agonist (BID), thus preventing the release of proapoptotic proteins from the mitochondria and favoring the anti-apoptotic pathway [58]. Importantly, this anti-apoptotic mechanism has also been highlighted in post-stroke exercise therapy [59], which suggests that both therapies share common pathways in their methods of conferring benefits to the ischemic brain.

### HIF-1α in Rehabilitative Effects Induced by Exercise and Ischemic Conditioning

Under hypoxic conditions, HIF-1α ensures adequate oxygen [60] and glucose supply [61] to the brain by inducing the transcription of VEGF, erythropoietin (EPO), and their respective receptors [62]. HIF-1α levels have also been observed to increase during exercise [63–66]. Furthermore, increases have been reported to be associated with remote ischemic preconditioning [67] and cerebral ischemia [68]. The present study concurs with these signs that HIF-1α levels positively correlate with beneficial effects. The present study further demonstrated the relationship between HIF-1α and regulation molecules such as BDNF, TrkB, and CREB. The cohort of rats with HIF-1α levels suppressed by HIF-1α inhibition saw increased infarct and greater brain dysfunction in ischemic rats after rehabilitation, which was associated with diminished neuroplasticity, synaptogenesis, angiogenesis, and signaling.

The present study indicated that RIPostC applied after ischemic stroke might be an effective alternative to therapeutic physical exercise after stroke. However, the contribution remains distant from full clinical integration, particularly in elderly patients, who typically have polypharmacy and various co-morbidities, such as hypertension, hyperlipidemia, and ischemic heart disease. In the context of these diverse medical issues, the use of conditioning should be determined carefully with consideration [69]. In our future study on rehabilitative strategy, it would be appropriate to consider the efficacy of blood plasma from young healthy donors with triggers and effectors of ischemic tolerance, activated by conditioning prior to the donation [70, 71].

## Conclusion

The present study found that both physical exercise and RIPostC following ischemic stroke improved stroke rehabilitation. As compared to post-stroke physical exercise, RIPostC was effective not only in acute reduction of infarct volume and neurological deficits, but also in various forms of chronic functional improvement, in association with increased neuroplasticity, angiogenesis, and synaptogenesis. These findings indicate that RIPostC may be an adequate alternative or adjunct for physical exercise in the setting of stroke rehabilitation. This is a valuable finding for the community of stroke survivors, as it could enable patients who have barriers to exercise to benefit from its rehabilitative mechanisms. Future investigations should focus on optimizing the initiation, dose, and duration of RIPostC to maximize therapeutic benefits, as well as exploring how physical and ischemic therapy could be implemented together to maximize rehabilitation.

## Data Availability

All raw data used in this manuscript are available on reasonable request.
