# Correction: Remote Ischemic Postconditioning vs. Physical Exercise After Stroke: an Alternative Rehabilitation Strategy?

**DOI:** 10.1007/s12035-025-04796-7

**Published:** 2025-03-03

**Authors:** Xiaokun Geng, Qingzhu Wang, Hangil Lee, Christian Huber, Melissa Wills, Kenneth Elkin, Fengwu Li, Xunming Ji, Yuchuan Ding

**Affiliations:** 1 Department of Neurology and China-America Institute of Neuroscience, Xuanwu Hospital, Capital Medical University, Beijing, 101149 China; 2 China-America Institute of Neuroscience, Luhe Hospital, Capital Medical University, Beijing, China; 3 Department of Neurology, Beijing Luhe Hospital, Capital Medical University, Beijing, China; 4 Department of Neurosurgery, Wayne State University School of Medicine, Detroit, MI USA; 5 Department of Research & Development Center, John D. Dingell VA Medical Center, Detroit, MI USA

**Correction: Molecular Neurobiology (2021) 58:3141–3157**

10.1007/s12035-021-02329-6

This is concerning with our article titled “Remote Ischemic Postconditioning vs. Physical Exercise After Stroke: an Alternative Rehabilitation Strategy?” published in volume 58 on page 3154 of Molecular Neurobiology before.

Recently, we identified an error in Fig. 10 Ang-2, where the internal reference β-actin (beta-actin), serving as a loading control, was mistakenly represented by the wrong blot.

After full consideration, we decided to make a correction on the figure based on the rigorous attitude of scholarship.

Fig. 10 with error being marked.
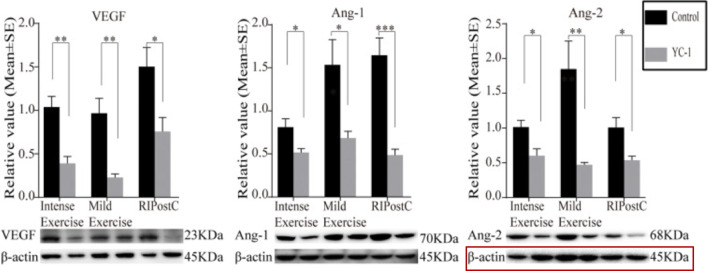


Herewith, the corrected images of Fig. 10.
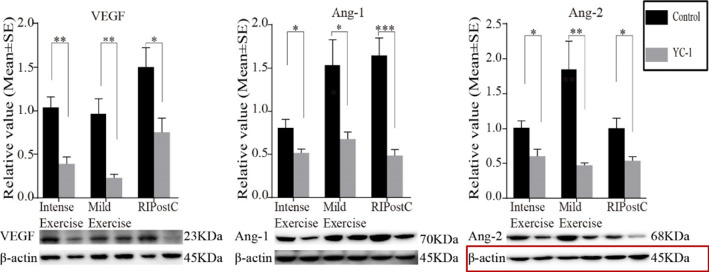


These corrections do not change the conclusions and text of the article. The authors apologize for any inconvenience caused to the readers and the editorial board for the Molecular Neurobiology.

The original article has been corrected.

